# Allergic Fungal Sinusitis: Ophthalmic Complications Due to the COVID-19 Pandemic and the Potential of Telemedicine

**DOI:** 10.7759/cureus.16458

**Published:** 2021-07-18

**Authors:** Stephen C Dryden, William I Evans, Paul J Percelay, Simon A Johnson, Mary E Hoehn

**Affiliations:** 1 Ophthalmology, The University of Tennessee Health Science Center, Memphis, USA

**Keywords:** ophthalmology, delayed presentation, delay, covid, allergic fungal sinusitis, orbit, delayed treatment, advanced disease

## Abstract

We report a case of a 26-year-old female who initially presented to an outside optometrist with complaints of proptosis and decreased visual acuity. Magnetic resonance imaging (MRI) obtained at that time was concerning for allergic fungal sinusitis. Unfortunately, the patient’s referral to ophthalmology was delayed due to the coronavirus disease 2019 (COVID-19) pandemic. On presentation to ophthalmology one year later, the patient had clinically deteriorated with significant visual and olfactory loss. She underwent emergent endoscopic sinus surgery by otolaryngology with histological analysis of the sinus debris confirming allergic fungal sinusitis. This is a unique case demonstrating the devastating impact that the COVID-19 pandemic had on patient care for an otherwise treatable condition. We propose the utilization of telemedicine networks as a way to prevent similar complications.

## Introduction

Allergic fungal sinusitis (AFS) is a noninvasive chronic reaction in immune-competent individuals to fungal deposition in the sinuses [1]. While approximately 17% of patients with AFS may experience ocular symptoms, these symptoms vary: proptosis, diplopia, tearing, telecanthus, vision loss, ophthalmoplegia, and ptosis [1-4]. While AFS is a benign, treatable disease, a delay in treatment can lead to a poorer outcome. We report the clinical findings of an advanced case of AFS in a patient with a delayed referral due to the coronavirus disease 2019 (COVID-19) pandemic and propose the utilization of telemedicine networks to prevent future complications from similar treatment delays.

## Case presentation

A 26-year-old female with no prior medical or ocular history presented to the clinic with a complaint of proptosis and decreasing visual acuity in both eyes, which she described as progressive for one year. The patient further endorsed anosmia, headache, and diplopia over a one-month period.

The patient reported that when she initially started to experience these symptoms, she presented to an optometrist. Her best-corrected visual acuity (BCVA) at that time was 20/25 OU. She was referred for a magnetic resonance imaging (MRI) study of her brain and orbits. The MRI at that time was concerning for allergic fungal sinusitis. The patient was non-urgently referred to an outside ophthalmologist for evaluation; unfortunately, she was lost to follow-up prior to referral due to the COVID-19 pandemic. No known attempts at telemedicine visits were made during this time.

On presentation one year later, the patient’s BCVA was 20/400 OD and hand motion OS. Both pupils were reactive with a left afferent pupillary defect (APD). Intraocular pressure was 11 mmHg OD and 12 mmHg OS. Extraocular movements were restricted: -3 abduction, -3 infraduction OD, and -1 abduction OS. Confrontational visual field testing was significant for a constricted field with suspected cecocentral scotoma in the right eye and was unable to be obtained in the left eye. The patient exhibited bilateral proptosis, right worse than left with a Hertel exophthalmometry reading of base 100 mm, 25 mm OD, and 24 mm OS. The remainder of the anterior segment exam was unremarkable. The patient’s dilated fundus exam was significant for a cup-to-disc ratio of 0.7 OD and 0.8 OS with nerve pallor OU. The fundoscopic examination was otherwise unremarkable. Optic nerve optical coherence tomography (OCT) demonstrated a global thickness of 62 OD and 52 OS with temporal thinning of both nerves OU. The patient was referred to the emergency room for further characterization with a computed tomography (CT) scan with maxillofacial and brain sequences, as well as otolaryngology and neurosurgery evaluation.

**Figure 1:**
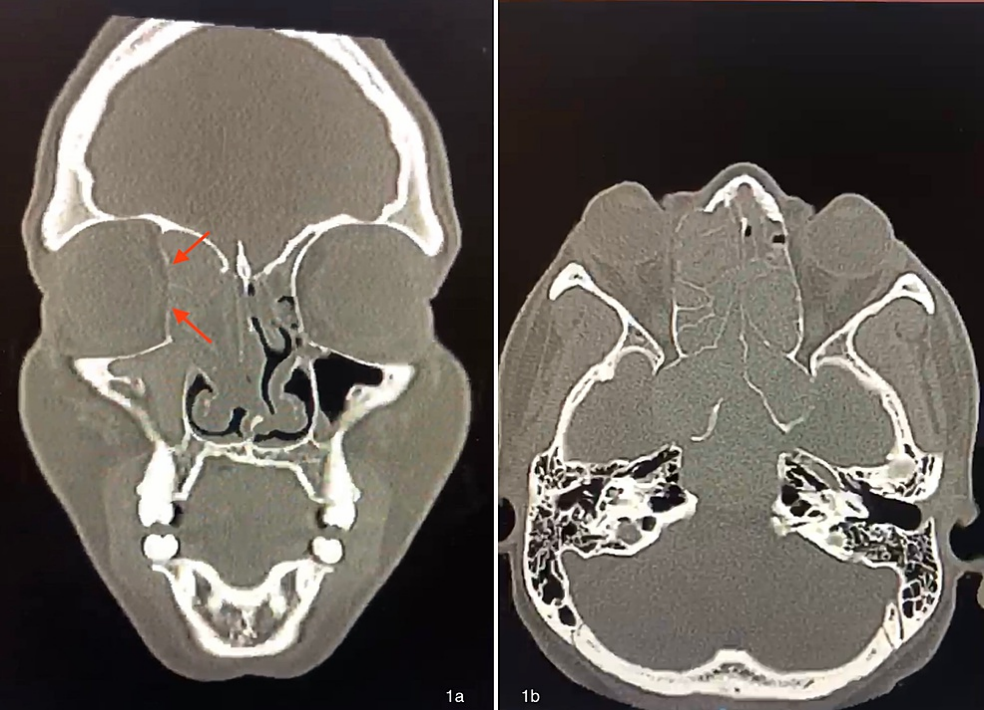
1a: Non-contrast coronal CT scan showing a bilateral pan sinus process with expansion causing thinning of the right medial orbital wall (arrows). 1b: Non-contrast axial CT scan showing bilateral ethmoid and sphenoid sinus process with posterior expansion with mass effect on the anterior cranial fossa and cavernous sinus and displacement of the pituitary gland

CT imaging was concerning for extensive bilateral sinus involvement and expansion, with thinning of the right medial orbital wall (Figure 1a). Additionally, there was noted to be superior and posterior expansion from the sphenoid sinus with resultant mass effect on the anterior cranial fossa and cavernous sinus and displacement of the pituitary gland, which closely abutted the optic chiasm/optic nerves (Figure 1b). There was no evidence of additional intracranial disease. Otolaryngology evaluated the patient and determined the patient needed emergent surgical debridement and corticosteroid therapy.

The patient underwent endoscopic sinus surgery with histological analysis of the sinus debris, which showed fungal hyphae entrapped in allergic mucin consistent with AFS. The patient was treated preoperatively with dexamethasone 8 mg TID for one day and discharged on a 10-day course of once-daily oral prednisone 10 mg. At her six-month follow-up examination, the patient’s vision had improved to 20/200 OD and counting fingers at five feet OS. Her intraocular pressure was normal, and she continued to demonstrate an APD OS. Her extraocular movements demonstrated -2 abduction OD and -1 abduction OS. Her proptosis showed an interval improvement, base 100 mm, 23 mm OD, and 22 mm OS. The remainder of her examination was stable.

## Discussion

AFS is a noninvasive chronic autoimmune reaction in immunocompetent individuals [1-3,5-8]. AFS is characterized by Type I (immunoglobulin E (IgE)-mediated) and Type III (IgG-mediated) immune reactions, chronic sinusitis, eosinophilia, and nasal polyposis [6]. AFS typically occurs in patients with atopic disease and asthma [2,6,9]. Involved sinuses contain a thick peanut-butter-like material with fungal elements and degraded eosinophils (Charcot-Leyden Crystals) [6,10]. Common ophthalmic manifestations at presentation are diplopia, proptosis, telecanthus, tearing, ophthalmoplegia, and vision loss due to expansile sinusitis and bony orbital erosion [1-2,8,10]. While uncommon, vision loss is a feared complication of AFS and can be due to optic neuritis from inflammatory exposure to fungal debris, retinal artery occlusion, or direct compression after bony erosion of the bony optic canal [8]. Treatment for AFS is a combination of systemic corticosteroids and surgical debridement [1,3,5,8-10].

While there are multiple reports of orbital involvement in patients with AFS, our case is unique in that there was a significant delay in referral due to the COVID-19 pandemic, which resulted in a poor visual outcome. Unfortunately, delayed presentation and treatment during the pandemic has been reported in the literature both domestically, with emergency department visits for severe ocular injuries, and abroad with ocular oncology and age-related macular degeneration leading to increased morbidity [11-13]. As the pandemic continues to evolve, it is imperative to examine referral pathways and barriers to care for all eye-related conditions to prevent future delays in care and associated poor visual outcomes.

Early intervention is key when considering progressive diseases, especially when dealing with the threat of irreversible damage. While the timeline for AFS progression is not well-established in the literature, there is evidence to support that time taken to treat the patient is one of the most important predictors of mortality. Some studies suggest treatment initiation within two weeks of symptom manifestation [14]. In this case, irreversible damage includes the loss of sight, making timely referral and intervention of utmost importance. Unfortunately, the urgency of this situation was not well-communicated between health care providers, and the patient was lost to follow-up for over a year during the COVID-19 pandemic.

Utilization of a robust telemedicine network is one possible solution that could have prevented the delay seen in our case. The COVID-19 pandemic has brought much attention to the topic of telemedicine, as social distancing and self-isolation have become prevalent. The recent relaxation of regulations and new reimbursement practices have compelled both ophthalmologists and patients to further consider this enticing option during these trying times [15-16]. New advances in technology have improved diagnostic accuracy and capabilities, with sensitivities for detecting certain diseases as high as 100% compared to in-office visits [17]. It is unknown if attempts were made at contacting the patient during the delay period; however, we suggest that a majority of patients with suspected AFS and active symptoms would want to pursue the opportunity of a telemedicine visit. In this case, the patient's worsening visual acuity would have warned against the ensuing impact of this disease and prompted an urgent surgical intervention. Telemedicine visits thus can serve as a way to monitor disease status and aid in determining when in-person clinical visits are absolutely necessary. This case is just one example of how telemedicine could have offered access to a patient before rapid progression caused deterioration of clinical status.

## Conclusions

Allergic fungal sinusitis is a noninvasive chronic autoimmune reaction in immunocompetent individuals and can be treated with a combination of surgical debridement and systemic steroids if caught early. Unfortunately, as demonstrated by our case, the COVID-19 pandemic resulted in a significant delay of presentation. In general, this delay can manifest as severe morbidity and mortality, and attention should be directed to best coordinate care in these unique times. Telemedicine offers one solution to combat these delays, and healthcare workers should remain attentive during scenarios where it can be best utilized.
